# Response to Surti *et al.*: ‘Pregnancy and liver transplantation’

**DOI:** 10.1111/j.1478-3231.2008.01922.x

**Published:** 2009-03

**Authors:** Christopher M Estes

**Affiliations:** Department of Obstetrics and Gynecology, Reproductive Health Services, University of Miami Miller School of MedicineMiami, FL, USA

To the Editor:

Surti and colleagues note in their comprehensive review of pregnancy following liver transplantation that the timing of pregnancy is linked to its success: the longer after transplantation, the lower the rate of obstetrical and graft-related complications (1–3). In order to achieve this goal, women must have access to reliable and effective contraception. While the AST consensus statement (4) recommends *against* the use of intrauterine devices (IUDs), the evidence cited for this is thin. It is based solely upon two cases reported in a single article in 1981 (5). Moreover, the risk of IUD-related pelvic infection in other immune-compromised individuals in not significantly increased compared with women with an intact immune system (6). Given these facts, when counselling women with a transplant, IUDs should be routinely offered as a first line method of contraception.

Intrauterine devices are used successfully and safely by women with many different types of transplants in my practice (and those of other experienced family planning providers). This letter is a call to the research community in the fields of organ transplantation and family planning to collaborate in an effort to investigate this important issue in more detail. Following this, appropriate, evidence-based recommendations could be made to aid clinicians and patients in selecting the best method of contraception. This will ensure that women with transplants remain healthy and are given the best opportunity to experience a healthy pregnancy.
